# Correspondence: On the bonding in ligand-protected gold clusters

**DOI:** 10.1038/s41467-017-01292-y

**Published:** 2017-11-15

**Authors:** Henrik Grönbeck

**Affiliations:** Department of Physics and Competence Centre for Catalysis, Chalmers University of Technology, Göteborg, SE-412 96 Sweden

## Introduction

Ligand-protected gold nanoparticles (AuNPs) have attracted scientific interest ever since Michael Faraday synthesized and studied optical properties of gold colloids^1^. The systems are widely used in sensing applications and represent examples in which electronic properties can be tuned by size and shape. For a long time, a detailed atomistic understanding of thiolate-protected AuNPs was missing due to experimental difficulties in synthesizing mono-dispersed AuNPs with high purity. However, following the complete determination of Au_102_(RS)_44_ in 2007^2^, a range of structures have been reported. A common motif in these geometries is a metal core protected by (RS)_*n*_Au_*m*_ units. Interestingly, this is the same motif as for self-assembled monolayers of thiolates on Au(111), which have (RS)_2_Au_1_ as protecting units^3,4^.

In a recent communication, Gao and co-workers^5^ suggest a scheme to rationalize the structures of ligand-protected AuNP. The authors adopt the language of nuclear physics and postulate two types of elementary building blocks (particles), namely Au$$_3^ + $$ and Au$$_4^{2 + }$$. The trimer (equilateral triangle) and tetramer (tetrahedron) are both two-electron closed-shell systems and are in ref. ^5^ associated with protons and tetraquarks, respectively.

Despite the fact that known ligand-protected AuNP can be decomposed according to the flexible scheme in ref. ^5^, the model is questionable with respect to the description of the electronic structure. Electronic properties of gold are determined mainly by delocalized 6*s* valence electrons. For bare gas-phase clusters, this is demonstrated by pronounced electronic shell closings that coincide with filled electronic shells for a spherical potential^6^. Moreover, bare and ligand-protected gold clusters develop localized plasmons^7^, which is an optical signature of delocalized valence electrons and the basis for sensing applications.

**Fig. 1:**
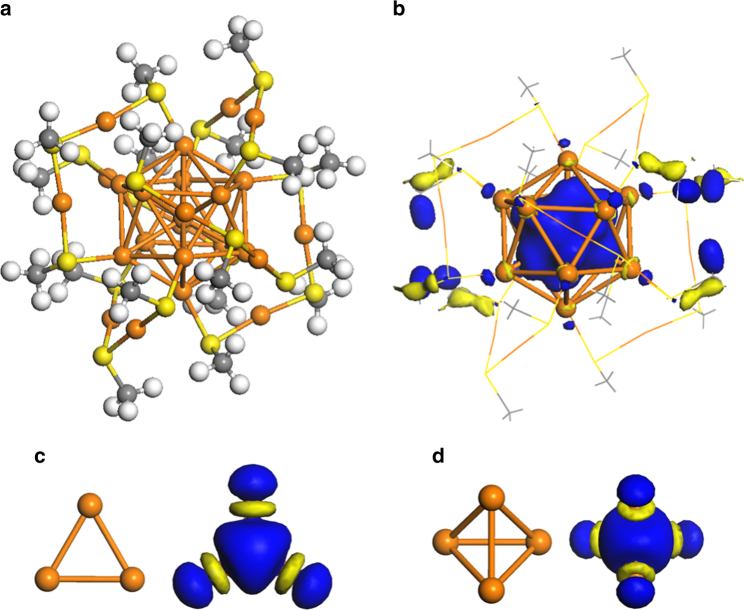
Structural models and selected Kohn-Sham orbitals. **a** Structural model of Au_25_(RS)$$_{18}^ - $$ with R being CH_3_. **b** The 1*S* orbital of Au_25_(RS)$$_{18}^ - $$. The protecting complexes are for clarity shown as lines. **d** The structure and 1*S* orbital of Au$$_3^ + $$. **d** The structure and 1*S* orbital of Au$$_4^{2 + }$$. Atomic color code: Au (orange), S (yellow), C (gray) and H (white)

The fundamental unit for gold in the condensed phase is the gold atom, and assigning a physical meaning to charged Au_3_ and Au_4_ in the AuNP is problematic. The coalescence of gold clusters induces bond re-hybridization, which makes the electronic configurations and properties of the initial constituents unimportant for the understanding of the final product. To make this clear, the case of Au_25_(RS)$$_{18}^ - $$ is taken as a simple example. The structure consists of an icosahedral core protected by six Au_2_(RS)_3_ complexes^8–10^, (Fig. 1). In ref. ^5^, the core is decomposed into two triangular and two tetrahedral units, where corner sharing between two units yields the thirteen-atom core. The optimized structure does not support this division. The average Au–Au distance in the core is 2.95 Å with a small s.d. 0.09 Å. If the structure was to be understood as fused Au$$_3^ + $$ and Au$$_4^{2 + }$$ units, the inter-unit distances would be larger than the intra-unit distances, in similarity with molecular crystals.

The structure of Au_25_(RS)$$_{18}^ - $$ is instead rationalized by electronic re-hybridization, where the gold atom is the basic building block. The Au_2_(RS)_3_ complexes are formally anions, which results in a closed eight-electronic shell for the core of Au_25_(RS)$$_{18}^ - $$ ^9^. The electronic configuration within the spherical jellium model is 1*S* ^2^1*P* ^6^, where the orbitals are delocalized over the entire cluster. The 1*S* orbital is shown in Fig. 1. This orbital is not linked to the 1*S* states of Au$$_3^ + $$ or Au$$_4^{2 + }$$ (also shown in the figure) but instead to linear combinations of atomic 6*s*-orbitals with a substantial weight on the central atom.

In ref. ^5^ it is suggested that the stability of the metal core in ligand-protected AuNP should be related to the closed-shell character and stability of Au$$_3^ + $$ and Au$$_4^{2 + }$$. However, highly charged metal clusters are generally unstable with respect to fragmentation. This holds, e.g., for the low energy Au$$_6^{2 + }$$ isomer discussed in ref. ^5^, which is unstable with respect to fragmentation into two Au$$_3^ + $$ units by 1.4 eV. Moreover, Au$$_4^{2 + }$$ is unstable with respect to fragmentation into two Au$$_2^ + $$ units by 0.2 eV. The stability of ligand-protected AuNP is consequently difficult to rationalize using Au$$_3^ + $$ and Au$$_4^{2 + }$$ as building blocks and without considering the bonds to the ligands.

The underlying reason why some sizes of ligand-protected AuNP are possible to crystallize and structurally characterize, while other sizes are difficult to characterize in this way is not yet known. The crystallization is often sensitive to the experimental conditions, which stresses kinetic effects. The facile formation and enhanced stability of some sizes is presumably a balance between electronic and structural factors. Closed electronic shells result in enhanced stability but do not appear to be an absolute condition. Common for all ligand-protected AuNPs are, however, delocalized valence electrons that are confined by the potential generated by the metal cores of the particles. Models that aim to link stability with structure should preferably account for this fundamental property.

## Methods

Density functional theory is used in an implementation with local basis functions and the gradient corrected exchange-correlation functional according to Perdew, Burke, and Ernzerhof (PBE)^11^. The Kohn–Sham orbitals are expanded with atomic numerical basis functions that are stored on radial grids centered at the atoms^12^. A double numerical basis set with polarization functions (dnp) is used for all atoms with a real-space cutoff of 5.0 Å. A semi-core pseudo potential with relativistic corrections^13^ is employed for Au to describe the interaction between the valence electrons (19 electrons) and the nuclei together with the inner-shell electrons.
